# Oral Hygiene Practice among 18-year-old Norwegian Adolescents Using Health Belief Model: A Cross-Sectional Study

**DOI:** 10.1055/s-0040-1719209

**Published:** 2021-12-01

**Authors:** Elwalid Fadul Nasir, Johnny Vu

**Affiliations:** 1 Research Department, Oral Health Centre of Expertise, Western Norway, Hordaland, Bergen, Norway; 2 College of Dentistry, King Faisal University, Alahsa, Kingdom of Saudi Arabia; 3 HEMIL institute College of Psychology, University of Bergen, Norway

**Keywords:** oral hygiene practice, brushing, flossing, health belief model, 18 years old, adolescence, Norway

## Abstract

**Objectives** The aim was to compare oral hygiene practice (brushing/flossing) among 18 years old from two regions, Hordaland County, Norway, and possible perceptional correlates using the Health Belief Model.

**Materials and Methods** The participants from six municipalities from the south district with high prevalence of dental caries to six municipalities from the rest of Hordaland county, with low prevalence of dental caries (control), using a web-based questionnaire. Statistical analyses: the Mann–Whitney U test was used and the *t* -test for independent samples. Bivariate and logistic regression analyses to examine associations.

**Results** A total of 416 people participated. The south district’s participants had lesser percentage brushing twice a day and flossing at least once a day, they significantly visited lesser the dental service, perceived more susceptibility to dental caries, and lower benefits of brushing/flossing compared with the controls. Girls (odds ratio [OR]: 0.34) who perceived higher severity of dental caries (OR: 1.86), higher self-identity (OR: 2.14), and lesser barriers to brushing (OR: 0.14) had higher odds to brushing twice a day compared with their counterparts. Girls (OR: 0.34) who perceived higher severity of dental caries (OR: 2.34), higher benefits (OR = 2.8), and lesser barriers to flossing (OR = 0.23) had higher odds to flossing at least once a day compared with their counterparts.

**Conclusion** South district’s participants significantly had some of risk factors to the recommended brushing/flossing practice compared with the control and these might help in explaining the difference in oral hygiene practice.

## Introduction

Positive oral health behaviors could be understood as behaviors related to removing dental plaque (oral hygiene, brushing/flossing), using fluoride toothpaste, adhering to a low cariogenic diet, and regular attendance to dental service. 1 2 It is therefore recommended twice-a-day tooth brushing and daily interdental flossing. 3

Adolescence (including 18 years old) is an important life period to promote favorable oral health perceptions and behaviors. As there seems to be some stability in health behaviors between adolescence and adulthood, which reflects lifestyles that are influenced by both life choices and life chances, 4 it is recommended to establish favorable oral hygiene habits at this age. 5

It is generally difficult to adopt or change certain behaviors, and there is often discrepancy between behavior and intention. 6 By assessing young peoples’ beliefs and perceptions toward oral health, we could possibly gain more knowledge and understanding of their oral health behavior. The Health Belief Model (HBM) was one of the first attempts to view health within the social context. It is a belief-based model and has been used to study a variety of health behaviors, including oral hygiene practices. 7 8 9

The HBM in the context of oral health suggests that a person would be more likely to comply with recommended oral hygiene behaviors (brushing/flossing) if the person believes that he/she is susceptible to oral diseases, that is, dental caries (perceived susceptibility) and that dental caries has serious consequences, that is, loss of teeth (perceived severity). A person who perceives lack of time, knowledge, or pain to practice oral hygiene is considered as having perceived barriers to the behavior, while if perceives that having good health as having perceived benefits from behavior. The conviction that a person can successfully fulfill the behavior (self-efficacy). 7 10

## Materials and Methods

The aggregated data of municipalities in Hordaland county showed that the municipalities that form the south district had a higher dental caries prevalence compared with the rest of the county and Norway as well. Oral hygiene is an important factor in dental caries experience. This study is a part of the project including other age groups (5 primary, 12 mixed, and 18 permanent dentition age groups). The 18 years old is a special age group in Norway as it is the starting age of payed dental public service. Therefore, the objective of this study was to assess any differences in oral hygiene practices (brushing/flossing) between 18 years old in the south district and the rest of Hordaland county, and possible explanatory cognitive factors using the (HBM).

The study included 12 municipalities, with all the six municipalities from the south district with high prevalence of dental caries as the exposure, and six municipalities from other districts of Hordaland county with low prevalence of dental caries (control), using a purposive sampling method based on the criteria of having the same number of 18 years old, and with lower prevalence of dental caries experience measured as dental caries experience (DMFT) obtained from reports of the Public Dental Health Service (PDHS), group data). The 18 years old were contacted through a text message to their private cellular phone for their acceptability and consenting. We used social security numbers obtained from the participants in the questionnaire to access their individual clinical records in (OPUS) medical record system for private and public dental clinics used in Norway in PDHS to collect information regarding individual dental caries experience (DMFT) and dental service utilization after written consent (in the questionnaire from each participant). A total of 613 agreed to participate, and 416 respondents completed the questionnaire, who were included in the analyses, giving a response rate of 37.5%. Of these, 350 gave consent to access information in their dental records. We obtained the approval for the study from the Norwegian Centre for Research Data. Informed consent was obtained from all participants; the confidentiality and safety of the information were secured in accordance with ethical and legal principles.

### Measures

The questionnaire included sociodemographic variables such as gender, country of origin, municipality, parents’ education, and employment.

Oral Hygiene Practices and Perceived Oral Health

We asked two questions to assess brushing/flossing, “how often did you brush/floss your teeth during the last week?.” Options were “not at all, once a week, every other day, once a day, and twice a day.” We measured self-administered fluoride by asking, “how frequent have you used fluoride rinse and/or tablets?” The use of the dental service assessed by asking, “how often have you visited a dentist over the past 5 years?.” Perceived good oral health by one statement: “I have good oral health.” One item measured fear/phobia related to dentist and syringe/needle.

### The Health Believe Model Constructs

Perceived severity of dental caries by using four items: “if I were to get dental caries, it would be very serious; if I were to get dental caries, it would hurt a lot; if I were to get dental caries, I would lose my teeth; and if I were to get dental caries, it would affect how I would feel in my daily life.” Perceived susceptibility for dental caries by analyzing the statements: “it is likely that I will get dental caries” and “within next year I will likely get dental caries.” Perceived benefits from oral hygiene practices by using statements for each practice: “brushing at least twice a day would prevent dental caries, my mouth feels better after I have brushed, flossing once a day prevents dental caries, and my mouth feels better after flossing.” Perceived barriers to oral hygiene practices by using items, respectively: “it hurts when I brush my teeth, my gums bleed when I brush my teeth, I forget to brush my teeth twice a day, I do not like the taste of toothpaste, it hurts when I floss, and my gum bleeds when I floss my teeth.” Self-identity toward oral health assessed through items: my teeth are an important part of who I am, I think of myself as a person who takes care of my teeth, it is important for me to have good dental health, and it is important for me to avoid cavities in my teeth” All items used a 5-point Likert’s scale ranging from “strongly disagree to strongly agree.” Regarding internal consistency of the scales measuring the constructs of the HBM and self-identity toward oral health, the Cronbach Alpha’s scores were as follows for self-identity toward oral health (0.83), perceived susceptibility to dental caries (0.88), and perceived severity of dental caries (0.70). For perceived benefit from flossing and brushing, the scores were 0.57 and 0.59, respectively, and for perceived barriers, for flossing and brushing were 0.61 and 0.60, respectively.

### Data Management and Analyses

We used Statistical Package of Social Sciences (SPSS) version 24 for data entry, management, and analyses. Nonparametric (the Mann–Whitney U) tests was used as an alternative to the *t* -test for independent samples, assessing the mean difference of the total scores of the HBM constructs. We performed bivariate, correlation, and logistic regression analyses to examine associations between oral hygiene behaviors, personal characteristics, and the HBM constructs.

## Results

**Fig. 1:**
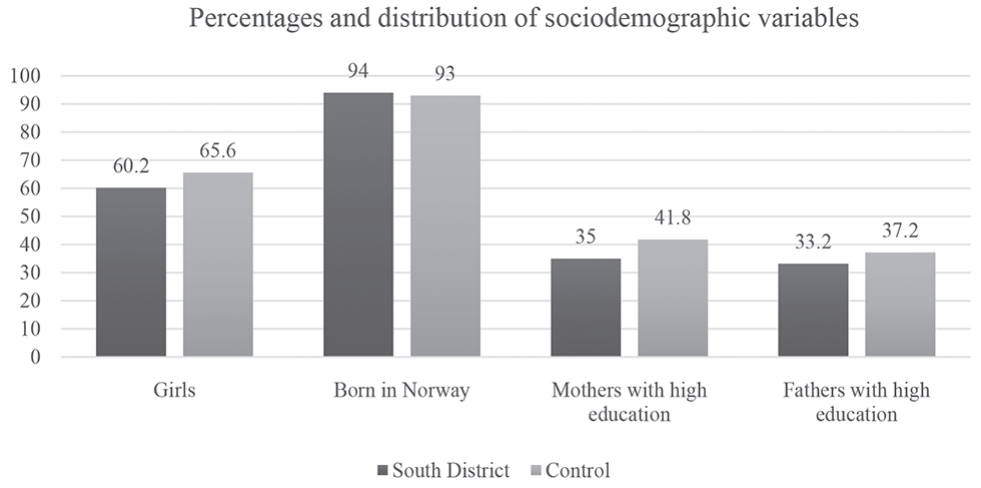
Percentage distribution of sociodemographic variables by the two groups.

Of the 416 respondents, 201 (48.3%) were from the south district. As presented in ( Fig. 1 ) in the total sample, there were more girls than boys 262 (63%). The large majority were born in Norway 389 (93.5%). About one-third reported that their mother or father had higher education. The control group had more girls, mothers, and fathers with high education compared with the south district.

The only significant difference between the two groups concerning oral hygiene behaviors was in visiting the dentist at least once a year during the last 5 years (79.1 vs. 89.8%, odds ratio: 2.3, 95% confidence interval: 1.3–4.0). In addition, participants from control group scored higher in toothbrushing, dental flossing, fluoride use, and perceiving good oral health ( Table 1 ).

**Table 1:** Percentages and frequency distribution of oral health behaviors by the two groups

Oral hygiene behavior	South district % ( *n* )	Control municipalities % ( *n* )	OR (95% CI)
Tooth brushing			
Twice a day	65.2 (131)	68.4 (147)	1.2 (0.8–1.7)
Dental flossing			
At least once a day	11.4 (23)	16.7 (36)	1.6 (0.9–1.7)
Fluoride use the past 5 years			
Yes	78.1 (157)	81.4 (175)	1.2 (0.8–1.9)
Afraid of the dentist			
Yes	24.9 (50)	21.5 (46)	0.8 (0.52–1.3)
Afraid of syringe needles			
Yes	37.8 (76)	35.8 (77)	0.9 (0.6–1.4)
Dental visits past 5 years			
At least once a year	79.1 (159)	89.8 (193) ^b^	2.3 (1.3–4.0)
Perceived oral health			
Good	72.5 (145)	73.5 (158)	1.1 (0.68–1.6)
Abbreviations: CI, confidence interval; OR, odds ratio. ^a^*p* < 0.05 ^b^*p* < 0.01 ^c^*p* < 0.00			

Regarding the self-identity and HBM constructs, there was a significant difference between the two groups as the participants from the south district scored higher in perceived susceptibility to dental caries, perceived lower benefits from oral hygiene practices, and from flossing compared with the controls. Other nonsignificant differences were also observed as participants from control municipalities perceived more severity of dental caries, benefit from brushing, less barriers toward oral hygiene, brushing and flossing, and higher oral health self-identity **(** Table 2 ).

**Table 2:** Mean score differences of Health Belief Model constructs between the two groups

Variables	Mean (SD)		Difference (SE)	95% CI
	South district	Control municipalities		
Severity	10.7 (0.2)	11.0 (0.2)	−0.27 (0.3)	−0.9 to 0.4
Susceptibility	6.0 (0.2)	5.5 (0.2)	0.55 (0.2) ^a^	0.1 to 0.9
Benefit brushing	9.1 (0.1)	9.3 (0.1)	−0.24 (0.1)	−0.5 to 0.0
Benefit flossing	7.4 (0.1)	7.9 (0.1)	−0.47 (0.2) ^b^	−0.8 to −0.2
Benefits OH	16.5 (0.2)	17.2 (0.2)	−0.72 (0.3) ^b^	−1.2 to −0.2
Barriers OH	13.6 (0.3)	13.3 (0.3)	0.31 (0.5)	−0.6 to 1.2
Barriers brushing	8.2 (0.2)	7.8 (0.2)	0.37 (0.3)	−0.3 to 0.9
Barriers flossing	5.4 (0.2)	5.5 (0.2)	−0.06 (0.2)	−0.5 to 0.4
Self-efficacy	17.1 (0.2)	17.4 (0.2)	−0.34 (0.3)	−0.9 to 0.2
Abbreviations: CI, confidence interval; OH, oral hygiene; SD, standard deviation; SE, standard error. ^a^*p* < 0.05 ^b^*p* < 0.01 ^c^*p* < 0.00				

Brushing twice a day was significantly related to perceiving high susceptibility, high benefits from brushing and oral hygiene, less barriers to oral hygiene and brushing, beside high self-identity. Those who reported brushing less than twice a day perceived more susceptible to dental caries, lesser benefiting from brushing, and oral hygiene practices. They also perceived more barriers toward oral hygiene, and brushing, and reported lesser self-identity toward oral health than those reporting brushing at least twice a day.

Flossing was significantly related to susceptibility, benefits flossing and oral hygiene, barriers oral hygiene and flossing, and self-identity. Those who reported flossing less than once a day felt more susceptible to dental caries, perceived lesser benefits from flossing and oral hygiene practices, perceived more barriers toward oral hygiene practices and flossing, and perceived lesser self-identity to oral health ( Table 3 ).

**Table 3:** Mean score differences of Health Belief Model constructs by brushing

HBM construct	Mean (SD) <2 times ≥2 times	Difference (SE)	95% CI
Brushing			
Severity	10.5 (0.3)/11.1 (3.4)	−0.57 (0.4)	−1.3 0.14
Susceptibility	6.4 (0.2)/5.4 (2.2)	0.9 (0.2) ***	0.47 1.39
Benefit brushing	8.6 (0.1)/9.4 (1.2)	−0.8 (0.1) ***	−1.09 −0.52
Benefit OH	15.9 (0.3)/17.3 (2.3)	−1.5 (0.3) ***	−2.03 −0.95
Barriers OH	15.9 (0.3)/12.2 (4.2)	3.6 (0.5) ***	2.74 4.52
Barriers brushing	10.2 (0.3)/l6.9 (2.8)	3.3 (0.3) ***	−2.03 −0.95
Self-efficacy	16.0 (0.3)/17.8 (2.5)	−1.8 (0.3) ***	−2.37 −1.27
Flossing			
Severity	10.7 (0.2)/11.7 (0.5)	−0.9 (0.5)	−1.86 0.03
Susceptibility	5.8 (0.1)/5.0 (0.3)	0.8 (0.3) *	0.18 1.42
Benefit flossing	7.4 (0.1)/9.0 (0.2)	−1.6 (0.3) ***	−2.05 −1.09
Benefit hygiene	16.6 (0.1)/18.5 (0.3)	−1.9 (0.4) ***	−2.65 −1.18
Benefit O Hygiene	13.9 (0.2)/10.3 (0.5)	3.6 (0.6) ***	2.39 4.87
Barriers O Hygiene	5.7 (0.1)/4.1 (0.3)	1.6 (0.3) ***	0.99 2.21
Self-efficacy	17.1 (0.1)/17.9 (0.4)	−0.8 (0.4)	−1.56 −0.01
Abbreviations: CI, confidence interval; HBM, health belief model; OH, oral hygiene; SD, standard deviation; SE, standard error. ^a^*p* < 0.05 ^b^*p* < 0.01 ^c^*p* < 0.00			

Hierarchical logistic regression brushing practices: the results indicated that the control variable gender in the first step, explained almost 10% of the variability in brushing behavior (Nagelkerke R-Square = 0.097). In the second step, inclusion of the predictor variables (visiting dentist and perceived oral health) explained 18.7% of the variability in brushing behavior (Nagelkerke R-Square = 0.187). In the third step, inclusion of HBM constructs (perceived severity of dental caries, susceptibility to dental caries, barriers and benefits of brushing, and self-identity oral health) increased the explained variability in brushing behavior to 42% (Nagelkerke R-Square = 0.42). The girls were almost three times likely to brush twice a day compared to the boys, three HBM constructs predicted brushing twice a day. Participants who perceived higher severity to dental caries were almost twice likely to brush twice a day ( *p* = 0.020). Those who scored higher self-identity were more than twice likely to brush twice a day ( *p* = 0.006). Those who perceived high barriers to brushing had decreased odds of brushing twice or more daily ( *p* = 0.000; Table 4 ).

**Table 4:** Brushing and flossing regressed by gender and Health Belief Model constructs

Variable	B	*p* -Value	OR	95% CI
Brushing				
Constant	0.8	0.125	2.14	
Gender				
Female			1	
Male	−1.1	0.000	0.34	0.20–0.58
Perceived severity of dental caries				
Low			1	
High	0.6	0.020	1.86	1.10–3.13
Perceived susceptibility of dental caries				
Low			1	
High	−0.3	0.29	0.73	0.41–1.31
Perceived self-efficacy oral health				
Low			1	
High	0.8	0.006	2.14	1.24–3.68
Perceived benefit from brushing				
Low			1	
High	0.5	0.064	1.64	0.97–2.77
Perceived barriers to brushing				
Low			1	
High	−1.9	0.000	0.14	0.83–0.25
Flossing				
Constant	−3.1	0.000	0.043	
Gender				
Female			1	
Male	−1.1	0.007	0.34	0.18–0.75
Perceived severity of dental caries				
Low			1	
High	0.9	0.011	2.34	1.22–4.49
Perceived susceptibility of dental caries				
Low			1	
High	0.1	0.74	1.11	0.59–2.11
Perceived self-efficacy oral health				
Low			1	
High	−0.0	0.97	1.001	0.50–2.02
Perceived benefit from flossing				
Low			1	
High	1.01	0.006	2.8	1.33–5.75
Perceived barriers to flossing				
Low			1	
High	−1.5	0.000	0.23	0.12–0.44
Abbreviations: CI, confidence interval; OR, odds ratio.				

Hierarchical logistic regression on flossing practice( Table 4 ) demonstrated that in the first step (gender) explained between 6% of the variability in flossing practices (Nagelkerke R-Square = 0.06). Adding behavioral variables in the second step (visiting dentist and perceived oral health) explained between 10% of the variability in flossing practices (Nagelkerke R-Square = 0.1). In step three, the inclusion of the HBM variables (perceived severity of dental caries, susceptibility to dental caries, barriers and benefits of flossing, and self-identity oral health) increased the explained variability in flossing practices to between 25% (Nagelkerke R-Square = 0.25). Gender, perceived severity, benefits from, and barriers to flossing were the strongest predictors. Girls were almost three times likely to floss at least once a day ( *p* = 0.007) compared with boys. Participants with higher perception of severity of dental caries had more than twice likelihood to floss at least once a day ( *p* = 0.011), and those with perceived benefits from flossing had three times likelihood to floss at least once a day ( *p* = 0.006), and those perceived less barriers to flossing had lesser odds to floss at least once a day ( *p* = 0.000).

## Discussion

The aim of this study was to assess and compare any differences in the oral hygiene behaviors (brushing and flossing) between the two groups and possible correlates using HBM.

In regard to personal characteristics, there was a slight difference between the two groups. There were higher percentages of girls and parents with high education among control participants. Of the total study sample, 66.8% reported brushing at least twice a day. This is clearly less than what was reported by World Health Organization (WHO) earlier among Norwegian girls (84%) and boys (65%), and Swedish girls (86%) and boys (78%). In Denmark, 86% of the girls and 76% of the boys with a significant difference between girls and boys. 11 The findings do not differ much between the Norwegian and other Nordic samples. However, a lower proportion among the participants could indicate that late adolescence time, a period with parent detachment where young people are increasingly becoming more independent from their parents. This period seems thus critical to promote brushing practice, which is essential in oral health care. 12 13 There was a slight difference with a higher percentage among control participants reporting brushing twice or more per day.

In regard to flossing practice, our finding—which is 14.2% of the total study sample reported flossing at least once a day—is slightly lower compared with the study of Norwegian adults in 2004, 14 where 16% of their sample of Norwegian adults reported daily flossing. Among 14-year-old Norwegian, half of the teenagers (54%) used dental floss and only 15% reported doing so daily. 15 There was also a slight difference with a higher percentage among control participants reporting flossing at least once a day.

These findings suggest that flossing practices compared with the recommendations seem to be less common than brushing practices among this sample of 18-year-old participants.

Various studies, as in our study, favored girls in relation to good oral hygiene practices. 16 17 18 The fact that adolescent females tend to have better oral hygiene practices (brushing) is in accordance the data from several countries gathered by the WHO. 19 These differences might be due to that females have higher health consciousness and are more inclined to visiting health professionals. 20 Another reason could be that females were found to possess better knowledge and oral health behavior-related self-efficacy. 21 Males have also been found to report more difficulties in performing oral hygiene behaviors, while females were reported to have more in control. 22

The only significant difference between the two groups was visiting the dental service in the last 5 years. Participants from control group visited more frequently. Studies supported the importance of regularly visiting the dental health service and oral health status. 23 Some studies related self-efficacy and visiting dental service. Luzzi and Spencer reported self-efficacy and past dental attendance were significant predictors of actual dental attendance. 24 Both higher brushing self-efficacy and dental visiting self-efficacy were found to be related to better brushing practices. 25 26 It could be an indication for the importance of dentists and dental hygienists as professionals to provide health education and promotion of positive oral health behaviors. 26 27 28

Relevant models from health psychology, such as HBM, seems promising to identify key beliefs to strengthen the favorable perceptions, reducing the barriers, affecting their attitudes, and increasing knowledge to form long-term and tailored oral health promoting and disease preventive measurements. 29 HBM has been supported by many studies as a suitable model for predicting health behaviors, in addition to being used in health education programs concerned with enhancing and promoting oral health behavior. 30 31 32 33

In terms of the HBM constructs, there were perceptional differences between the two groups as participants from control perceived more susceptibility, benefits from oral hygiene, and flossing. They also perceived higher self-efficacy, lesser barriers toward oral hygiene, and brushing. After controlling for gender, self-efficacy toward oral health, perceived severity of dental caries, and perceived barriers to brushing significantly predicted brushing practice. Whereas perceived severity, barriers to, and benefits from flossing were strong predictors of flossing practices. These factors predicted brushing and flossing practices, respectively.

Self-efficacy toward oral health significantly predicted brushing practices in the present study, which is in accordance with the literature. Various studies have reported self-efficacy as the most significant factor related to oral hygiene practice. In a study among first-year medical students, having better self-efficacy toward oral health related with better oral health behavior. 22 A study among pregnant women as well as among children’s guardians found self-efficacy as the only factor related to oral hygiene practice. 34 35 In a study among first-year medical students, having better self-efficacy toward oral health related with better oral health behavior 24 . Increasing oral health self-efficacy should be considered as an important factor in maintaining and promoting better oral hygiene practice. In our findings, participants from control group had higher perceived severity than those from the south district, in addition to its significant relation with predicting both oral hygiene practices (brushing/flossing). The strength of perceived severity as a predictor for brushing practices is also supported by many studies that found perceived severity significantly predicted tooth brushing frequency. Kasmaei et al found that perceived severity plays an important role in adapting a desirable health behavior among young adolescents, Anagnostopoulos et al reported that perceived severity of oral diseases was related to increased toothbrushing frequency, and Solhi et al observed the correlation between the performance of brushing/flossing and perceived severity. 8 29 36 Increased knowledge and perceiving oral health-related problems as more severe have been found to associate with perceiving more benefits from oral health behaviors and less barriers to brushing.

Our results showed a significant difference in perceived benefits between the two groups with control participants scoring higher. Perceived benefits were also significantly related to flossing. Many studies in the literature have reported this significant relation. Solhi et al, Charkazi et al, and Schluter et al reported a correlation between the performance of brushing/flossing and benefits. 29 37 38 It was observed that the control participants perceived less barriers to oral hygiene practice compared with participants from the south district. Perceived barriers were also found as significant predictors of brushing and flossing. Studies that had used the framework of the HBM have in general found support for perceived barriers to be the only predictor of oral hygiene practices. For instance, among Iranian students, perceived barriers were the only core construct that explained the oral health behavior, 39 and similarly in another study, perceived barriers were the only core construct of the HBM that explained both flossing and brushing behaviors among Australian dental patients. 18 Another study among Iranian female students grade four, partially supported that perceived barriers (perceived psychological barriers) predicted oral hygiene practices. 8 Many studies reported barriers as predictors to oral hygiene practice. 40 41 42 These findings suggest that knowledge and fear appeal could possibly be used to increase the perception of severity from oral diseases, to increase the perception of the benefits and reduce strength of the perceived barriers. In relation to oral health, the exact nature of the relationship between perceptions and behaviors is complex.

## Conclusion

The findings showed that the perceptional differences between the two groups might explain the difference in oral hygiene practice (brushing/flossing). Adjusted analysis demonstrated that self-efficacy toward oral health, perceived severity of dental caries, and perceived barriers to brushing significantly predicted brushing practice, whereas perceived severity, barriers to and benefits from flossing, predicted flossing. It is therefore important that these factors are assessed in the targeted population when planning public health campaigns. This is possibly even more important for late adolescence. These factors might be used as driving elements in maintaining and promoting the oral hygiene practice. The results might be used in designing health prevention and promotion strategies to maintain better oral health for the adolescents in the south district.

The understanding of oral health-related perceptions and self-efficacy toward oral health, and the cognitive and psychological processes behind informed personal decisions to adopt oral health-related behavior are important parts in the planning of interventions and measurements directed at oral disease prevention and oral health promotion. 43 Instability in oral health perceptions from adolescence to young adulthood was related with no recommended oral health behavior, poorer self-rated oral health, and poorer oral health status. 44

### Limitations

All the results must be cautiously interpreted as this is an observational study with its known limitations. One weakness to be mentioned is the DMFT data that was extracted from the participant´s records (secondary data), which lacks standardization and calibration of the dentists and dental hygienists that have made the registrations. Another limitation might be self-reported information about the behaviors and HBM model constructs (information bias). This might have had social-desirability bias and recall bias. The selection bias is also to be considered as those who did not participate might have had different characteristics and opinions from the actual participants. The small sample size might have affected the level of precision and the generalizability of the study.
